# In vitro bioaccessibility of macular xanthophylls from commercial microalgal powders of *Arthrospira platensis* and *Chlorella pyrenoidosa*

**DOI:** 10.1002/fsn3.2150

**Published:** 2021-02-16

**Authors:** Cristina Tudor, Elena Cristina Gherasim, Francisc Vasile Dulf, Adela Pintea

**Affiliations:** ^1^ University of Agricultural Sciences and Veterinary Medicine Cluj‐Napoca Romania

**Keywords:** bioaccessibility, carotenoids, dietary supplements, microalgae, simulated digestion, *Spirulina*

## Abstract

The bioaccessibility of the major carotenoids present in two commercial microalgal supplements in powder form was investigated through a standardized in vitro digestion method. The dried biomass of *Arthrospira platensis* contained β‐carotene (36.8 mg/100 g) and zeaxanthin (20.8 mg/100 g) as the main carotenoids as well as a high content of saturated fatty acids (61% of total fatty acids), whereas that of *Chlorella pyrenoidosa* was rich in lutein (37.8 mg/100 g) and had a high level of unsaturated fatty acids (65% of total fatty acids). In the case of the latter, lutein bioaccessibility was not statistically enhanced after the replacement of porcine bile extract with bovine bile extract in the in vitro digestion protocol and after the addition of coconut oil (17.8% as against to 19.2% and 19.2% vs. 18.5%, respectively). In contrast, the use of bovine bile extract along with co‐digestion with coconut oil significantly enhanced the bioaccessibility of zeaxanthin from *A. platensis*, reaching the highest bioaccessibility of 42.8%.

## Graphical abstract

The content (HPLC‐PDA) and the bioaccessibility of the major carotenoids present in two commercial supplements of *Arthrospira platensis* and *Chlorella pyrenoidosa* was investigated through a standardized in vitro digestion method, in the presence or absence of coconut oil and porcine or bovine bile extract. *A. platensis* contained β‐carotene and zeaxanthin as the main carotenoids and a high content of saturated fatty acids, whereas *C. pyrenoidosa* was rich in lutein and had a high level of unsaturated fatty acids. The use of bovine bile extract along with co‐digestion with coconut oil significantly enhanced the bioaccessibility of zeaxanthin from *A. platensis*, while lutein bioaccessibility was slightly enhanced in the presence of bovine bile extract but no significant effect was observed after the addition of coconut oil.

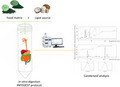

## 1 INTRODUCTION

Often considered the food sources of the future (Torres‐Tiji et al., 2020), microalgae can provide an abundance of essential nutrients to human health (Koyande et al., 2019). Apart from high‐quality proteins (Chronakis & Madsen, 2011), the edible biomass of microalgae is comprised of carbohydrates, long‐chain polyunsaturated fatty acids, vitamins, minerals, and other bioactive compounds such as carotenoids (Henríquez et al., 2016; Schweiggert & Carle, 2016). Lately, the trend toward carotenoid consumption increased after a plethora of publications suggested their positive effects in conditions related to oxidative and inflammatory stress, cancer, cardiovascular disorders, and ocular diseases such as age‐related macular degeneration, cataracts, and retinitis pigmentosa (Fiedor & Burda, 2014; Kaulmann & Bohn, 2014). Lutein and zeaxanthin are known to be responsible for maintaining ocular health by reducing the risk of visual impairment over time (Johnson, 2014) and have also been associated with cognitive function (Hammond et al., 2017), thus finding alternative and bioaccessible food sources of these beneficial xanthophylls constitutes an important research direction.

Several microalgae such as *Chlorella* sp., *Arthrospira* sp., and *Dunaliella* sp. can accumulate high amounts of carotenoids, particularly β‐carotene and xanthophylls (Cha et al., 2012; Grosshagauer et al., 2020; Tang & Suter, 2011). The incorporation of microalgae in novel food formulations is currently limited due to their unappealing sensory properties and human consumption of these dietary supplements is usually via the ingestion either of a compact form (as tablets, capsules, and pastilles) or a powdered form (as a food complement). Microalgal biomass for human consumption is mostly available in the form of dried powder (de Farias Neves et al., 2019), usually marketed as commercial microalgal powder. Microalgae cultivated in open ponds or in closed photobioreactors are harvested, processed, and extracted in order to obtain the valuable powder used not only in the food industry, but also in feed, nutraceutical, and pharmaceutical fields (Benedetti et al., 2018).

It has been generally acknowledged that the bioaccessibility of some carotenoids is higher from animal‐based foods than from plant sources (Chacón‐Ordóñez et al., 2018), but data are still insufficient as concerns microalgal sources. Carotenoid release from the structural organizations in which they are embedded in different food matrices represents one of the major factors that affect bioaccessibility. Regardless of the fact that food processing may result in the loss of a certain amount of bioactive compound, disintegration of the matrix can have a beneficial impact on carotenoid liberation and bioaccessibility (Schweiggert & Carle, 2015). In the case of commercial microalgae supplements, the cell wall is usually disrupted by the manufacturer during the drying process of the powders to ensure a better nutrient assimilation (de Farias Neves et al., 2019; Villarruel‐Lopez et al., 2017), and in some species such as *Arthrospira platensis* (more commonly known as *Spirulina*), additional processing is not required owing to the absence of cellulose in the cell wall (Hosseini et al., 2013).

Even though plenty of research is available regarding the nutrient profile of some edible microalgal products, data on carotenoid bioaccessibility and intestinal absorption are scarce and focused only on a few of the multitude microalgae species (Bernaerts et al., 2020; Cha et al., 2011, 2012; Gille et al., 2015, 2017, 2018; Granado‐Lorencio et al., 2009). For instance, the existing literature on *C. pyrenoidosa* (a unicellular green microalga) is limited and mainly centered on the positive effects upon administration (Merchant et al., 2002; Nakano et al., 2007, 2009). Also, as far as we are aware, there are no publications with respect to carotenoid bioaccessibility from this edible microalga. In contrast, the more popular *A. platensis* (a multicellular blue‐green microalga) has been the focus of a great number of investigations (Gershwin & Belay, 2007; Seyidoglu et al., 2017; Yu et al., 2012) over the past decades, but even in this case information regarding carotenoid gastrointestinal fate is sparse. Given that these commercially available supplements are highly promoted and consumed throughout the world not only by athletes (mainly for protein intake enhancement) but also by the population at large, it is clear that more studies on the bioaccessibility of bioactive compounds from microalgae are required in order to fully understand their benefits.

In this context, we sought to evaluate the bioaccessibility of the major carotenoids from *A. platensis* and *Chlorella pyrenoidosa*, two of the most consumed microalgal supplements (Koyande et al., 2019; Pulz & Gross, 2004), using an internationally recognized in vitro digestion protocol (Minekus et al., 2014). As a means to improve carotenoid bioaccessibility, the addition of organic cold‐pressed coconut oil (*Cocos nucifera* L.) along with the two supplements in the in vitro digestion protocol was studied and the use of both porcine and bovine bile extracts in the intestinal phase was tested.

## 2 MATERIALS AND METHODS

### 2.1 Materials

All chemicals and reagents were of analytical grade and ultrapure water (18 MΩ cm resistance) treated in a Milli‐Q water purification system was used throughout the experiments. α‐Amylase from human saliva (A1031), pepsin from porcine gastric mucosa (P6887), pancreatin from porcine pancreas (P7545), porcine bile extract (B8631), and bovine bile extract (B3883) were purchased from Sigma‐Aldrich. Carotenoid standards β‐carotene, lutein, and zeaxanthin (purity ≥ 98%, ≥95% and ≥98%, respectively) were acquired from Extrasynthese.

The organic powders of *A. platensis* and *C. pyrenoidosa* and cold‐pressed coconut oil (*C. nucifera* L.) were bought from a local health‐food store and stored at room temperature in their original packaging until use. The two organic microalgal powders were purchased from the same company (China was specified as the country of origin), and the nutritional values listed on the labels are shown in Table 1. The daily recommended dosage on the supplier's label was 3–10 g (1–3 spoons) of *A. platensis* and 2–5 g (1–2 spoons) of *C. pyrenoidosa*.

**TABLE 1:** Composition of *Arthrospira platensis* and *Chlorella pyrenoidosa* organic powders as indicated on the labels

	*Arthrospira platensis*	*Chlorella pyrenoidosa*
	Nutritional value for 100 g powder	
Energy	342.5 kcal	373.9 kcal
Total fat	0.76 g	2.61 g
*of which saturated*	0.42 g	0.61 g
Total carbohydrates	13.98 g	28.44 g
*of which sugars*	0.10 g	1.16 g
Fibers	4.20 g	9 g
Proteins	67.85 g	59.17 g
Salt	0.90 g	0.064 g

### 2.2 Carotenoid extraction from starting materials

Extraction was performed at room temperature and under dimmed light. Approximately 0.4 g of each organic microalgal powder was weighed (in triplicate) in 15‐ml tubes, and 4 ml of hexane:acetone (1:1, v/v) was added. The tubes were homogenized using a vortex mixer for 1 min and centrifuged at 4,800 *g* (Eppendorf 5810 R, Eppendorf AG) for 10 min to speed up phase separation. The procedure was repeated with 4 ml hexane until the supernatant was colorless. The carotenoid‐containing extracts were combined, reduced to dryness at 35°C with the use of a rotary evaporator (Heidolph MR Hei‐End) and stored at −20°C until HPLC‐DAD analysis.

### 2.3 HPLC‐DAD analysis of carotenoids from starting materials and micellar fraction

The extracts were dissolved in a known volume of ethyl acetate and filtered (0.20 μm PTFE filter) in amber vials. The HPLC system (Shimadzu Corporation) was equipped with a SPDM20A diode array detector and an YMC C30 reversed phase column (250 × 4.6 mm i.d., 5 µm particle size). Carotenoid separation was performed applying a gradient elution with methanol/tert‐butyl methyl ether/water (83:15:2, v/v/v) (solvent A) and tert‐butyl methyl ether/methanol/water (90:8:2, v/v/v) (solvent B) in the following conditions: 0 min 0% solvent B, 20 min 0% B; 150 min 82% B; 152 min 0% B, where after the column was equilibrated for 10 min. The flow rate was 0.8 ml/min, and the injection volume was 20 μl. Individual carotenoids were identified by comparing their retention time, elution order on C30 column, UV‐Vis spectra (*λ*_max_, spectral fine structure (%III/II) with those of the available standards (β‐carotene, lutein, zeaxanthin and β‐cryptoxanthin)) and with literature data. Quantification of the three major carotenoids was performed using external ten‐point calibration curves constructed in the range 1–100 μg/ml. The correlation coefficients were as follows: *R* ^2^ = .9912 (β‐carotene), *R* ^2^ = .9991 (lutein), and *R* ^2^ = .9996 (zeaxanthin).

### 2.4 Lipid extraction from starting materials

Lipid extraction was performed according to the method described by Folch et al. (1957). In short, approximately 2 g of each microalgal biomass was weighted (in triplicate) and 10 ml methanol was added. After a vigorous homogenization, 20 ml of chloroform was added and the mixture was again homogenized. After filtration, the residual biomass was re‐extracted using a mixture of chloroform:methanol (2:1, v/v). KCl 0.88% was added to the combined extracts in a separating funnel so as to achieve a ratio of 8:4:3 (v/v/v) chloroform:methanol:potassium chloride. The hypophase, that is, the lipid‐containing chloroformic phase, was dried with anhydrous sodium sulfate, evaporated to dryness at 35°C with a rotary evaporator (Heidolph MR Hei‐End) and stored at −20°C prior to GC‐MS analysis.

### 2.5 Fatty acid GC‐MS analysis of coconut oil and microalgae lipids

By using the acid‐catalyzed transesterification method (Christie, 1989), fatty acid methyl esters (FAMEs) were determined employing a PerkinElmer Clarus 600T GC‐MS (PerkinElmer, Inc.) equipped with a SUPELCOWAX 10 (Supelco Inc.) capillary column (60 m × 0.25 mm i.d., 0.25 μm film thickness) and helium as carrier gas (0.8 ml/min flow rate). The initial oven temperature (140°C) was increased by 7°C/min to 220°C and kept 23 min at 220°C. The injection volume was 0.5 μl (split ratio of 1:24), and the injector temperature was set at 210°C. The positive ion electron impact (EI) mass spectra was recorded at an ionization energy of 70 eV and the trap current of 100 μA, with the source temperature of 150°C and a scanned mass range of 22–395 m/z. FAMEs were identified twofold: (a) by correlating the retention times with those of known standards (37 component FAME Mix, SUPELCO, Art. No. 47885‐U) and (b) by comparing their mass spectra with the data provided by the MS database (NIST MS Search 2.0). Fatty acids were expressed as percentage (%) of total fatty acids. Analyses were carried out in triplicate, and the mean values were reported.

### 2.6 Simulated digestion model

The simulation of the gastrointestinal passage was performed according to Minekus et al. (2014). The two organic powders were subjected to an in vitro digestion protocol consisting of three phases, that is, oral, gastric, and small intestinal phases. The effect of an added dietary lipid source on the bioaccessibility of carotenoids from *A. platensis* and *C. pyrenoidosa* was investigated through the inclusion of organic coconut oil (5%) in the oral phase. Moreover, the influence of the bile source (porcine or bovine) on carotenoid bioaccessibility was analyzed.

Briefly, 1 g of organic powder in 2.25 ml water was mixed with 2 ml simulated salivary fluid (SSF), 0.6 ml α‐amylase in SSF (75 U/ml in final digestion mixture), 162.5 μl CaCl_2_ (0.03 M), and 487.5 μl water. After a short homogenization, the mixture (pH 7) was incubated at 37°C for 2 min (150 orbital shakes/min) in a shaking water bath (Memmert GmbH + Co. KG). The oral bolus was combined with 4.2 ml simulated gastric fluid (SGF), 1 ml porcine pepsin in SGF (2,000 U/ml in final mixture), and 32.5 μl CaCl_2_ (0.03 M). The pH was adjusted to 3.0 with HCl (1 M), water was added to achieve a final volume of 13 ml, and the mixture was incubated under agitation for 2 hr. The gastric chyme was diluted with 6.4 ml simulated intestinal fluid (SIF), 2 ml pancreatin in SIF (based on the activity of trypsin, 100 U/ml in final digestion mixture), 2 ml bile salts in SIF (10 mM in final digestion mixture), and 260 μl CaCl_2_ (0.03 M). After homogenization, the pH was adjusted to 7.0 with NaOH (1 M), water was added to a volume of 26 ml, and the final digestion mixture was incubated for 2 hr. The resulting digesta was centrifuged for 60 min at 4°C (4,800 *g*; Eppendorf 5810 R), and an aliquot of the micellar phase was filtered (0.2 μm nylon filter) and stored at −80°C. Carotenoid extraction was described in an earlier study (Tudor et al., 2020).

Carotenoid bioaccessibility (%) was calculated as the concentration of carotenoids in the micellar phase versus the initial carotenoid concentration in the microalgae powders.

### 2.7 Statistical analysis of data

All analyses were carried out in triplicate and reported as mean ± standard deviation (*SD*). Data interpretation was performed using unpaired *t* test with Welch's correction of Graph Pad Prism, Version 6.0 (Graph Pad Software Inc.). *p* values < .05 were considered statistically significant.

## 3 RESULTS AND DISCUSSION

### 3.1 Characterization of the investigated microalgal powders

**FIGURE 1:**
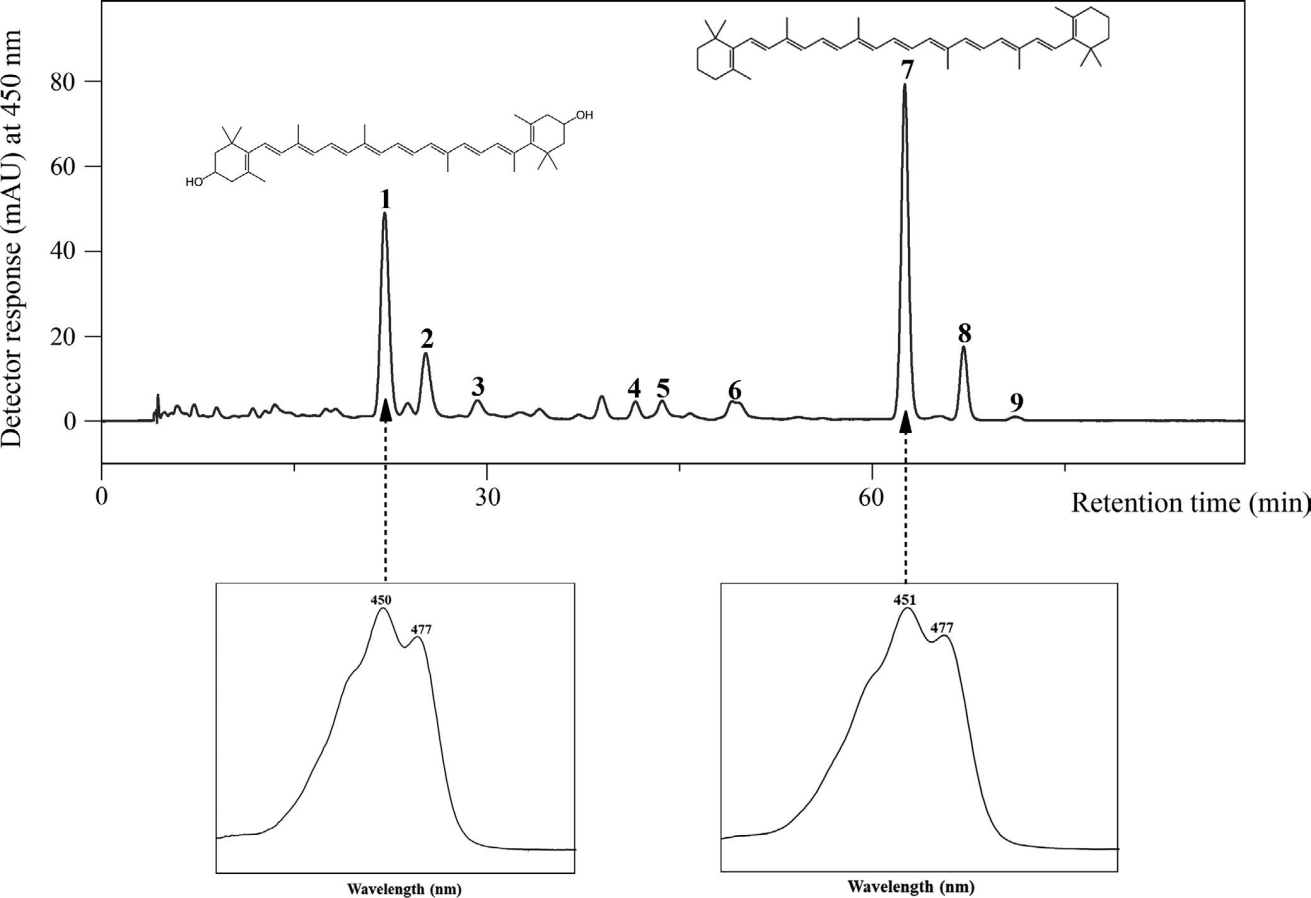
HPLC‐DAD chromatogram of carotenoids in *Arthrospira platensis* extract. Peaks: 1, zeaxanthin; 2, chlorophyll a; 3, chlorophyll a isomer; 4, β‐cryptoxanthin; 5, echinenone; 6, 13‐*cis*‐β‐carotene; 7, *all*‐*trans*‐β‐carotene; 8, 9‐*cis*‐β‐carotene; 9, α‐carotene. Peaks 2–7 and 8–9 were tentatively identified based on the spectral characteristics and elution order previously reported in the literature

**FIGURE 2:**
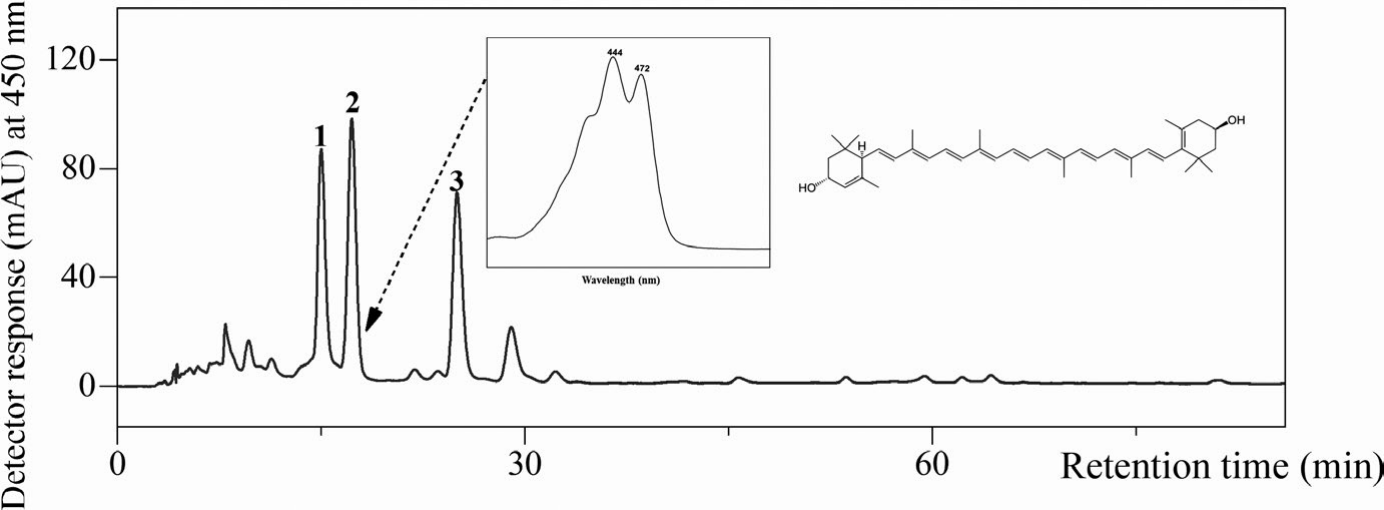
HPLC‐DAD chromatogram of carotenoids in *Chlorella pyrenoidosa* extract. Peaks: 1, unidentified chlorophyll; 2, lutein; 3, unidentified chlorophyll

The two edible microalgae *A. platensis* and *C. pyrenoidosa* were characterized in terms of fatty acid profile and major carotenoid content (Table 2). As previously reported in the literature, the content and composition of pigments varies greatly not only between different species but also between studies focused on the same microalgal strain. In our study, the major carotenoid in *A. platensis* powder was β‐carotene (36.75 ± 0.63 mg/100 g), followed by zeaxanthin (20.78 ± 0.49 mg/100 g) (Figure 1), while the dried biomass of *C. pyrenoidosa* contained a considerable amount of lutein (37.75 ± 0.36 mg/100 g) (Figure 2). Other available publications investigating the carotenoid content of *A. platensis* show a great variation. For example, the content of β‐carotene obtained in the present study was within the range of Grosshagauer et al. (2020) (33.5–231.6 mg/100 g) but much lower than the amount reported in another *Spirulina* supplement (211 mg/100 g) (Tang & Suter, 2011). More consistent to our findings, in a study investigating the carotenoid content of six commercial *Spirulina platensis* dietary supplements from Czech Republic (three in a powder form and three as pills), β‐carotene was found in the range of 8.69–103.7 mg/100 g and zeaxanthin between 2.78 and 60.6 mg/100 g (Hynstova et al., 2017). All the few existing publications focusing on the carotenoid content of *C. pyrenoidosa* (Fan et al., 2015; Inbaraj et al., 2006; Wu et al., 2007) reported lutein as the major carotenoid but in a higher amount than that determined in the current study, 39.33–124.01 mg/100 g (Fan et al., 2015) and 125,034.4 μg/g (Inbaraj et al., 2006). This wide variation in pigment composition and content within studies targeting the same microalgal strain occurs most probably due to a combination of various factors such as the different geographical origin, cultivation conditions, and processing techniques (Hosseini et al., 2013). Knowing that an “established” profile for each strain is unattainable, the analysis of microalgal bioactive compounds proves a valuable tool in monitoring the quality of these dietary supplements readily available to the general population.

**TABLE 2:** Characterization of *Arthrospira platensis* and *Chlorella pyrenoidosa* in terms of fatty acid composition and major carotenoid content

Fatty acid (% of total)^a^	*Arthrospira platensis*	*Chlorella pyrenoidosa*
Palmitic acid (16:0)	60.74 ± 1.52	31.22 ± 0.41
*cis*‐Hypogenic acid (16:1 n−9)	3.02 ± 0.30	1.23 ± 0.10
7,10‐Hexadecadienoic (16:2 n−6)	nd	17.83 ± 0.45
Stearic acid (18:0)	0.34 ± 0.06	3.43 ± 0.23
*cis*‐Vaccenic acid (18:1 n−7)	2.62 ± 0.37	1.53 ± 0.25
Linoleic (18:2 n−6)	19.24 ± 0.47	36.02 ± 0.55
γ‐Linolenic (18:3 n−6)	14.04 ± 0.55	0.58 ± 0.08
α‐Linolenic (18:3 n−3)	nd	8.16 ± 0.20
Σ SFA	61.07 ± 1.58	34.65 ± 0.64
Σ MUFA	5.64 ± 0.67	2.76 ± 0.35
Σ PUFA	33.28 ± 1.02	62.60 ± 1.27
Carotenoids (mg/100 g)^a^		
Lutein	nd	37.75 ± 0.36
Zeaxanthin	20.78 ± 0.49	nd
β‐Carotene	36.75 ± 0.63	nd

^a^ Mean ± *SD* (*n* = 3); nd = not detected, SFA represents saturated fatty acids; MUFA represents monounsaturated fatty acids; PUFA represents polyunsaturated fatty acids.

The gravimetric determination of total lipids revealed a content of 0.73 g lipids/100 g powder (*A. platensis*) and, respectively, 2.70 g lipids/100 g powder (*C. pyrenoidosa*). With respect to the fat content, the experimental values were in agreement with the labeled ones (Table 1). Regarding the fatty acid composition, the dried biomass of *A. platensis* used in this study contained 61.07% saturated fatty acids (SFA), whereas that of *C. pyrenoidosa* 34.65%, of total fatty acids content (Table 2). These values were slightly higher than those of Ötleş and Pire (2001), which reported the content of saturated fatty acids ranging between 51.64% and 55.72% in three commercial powders of *S. platensis* and between 24.73% and 33% in three commercial powders of *C. pyrenoidosa*. The same authors identified in *S. platensis* a fatty acid profile similar to our findings, with palmitic acid (16:0) as the major fatty acid (42.30%–46.07%), along with high amounts of γ‐linolenic (18:3 n−6) (8.87%–21.73%) and linoleic (18:2 n−6) (16.18%–17.43%) acids. As regards, *C. pyrenoidosa*, a different fatty acid profile, was observed in our case in which the predominant fatty acids were linoleic (18:2 n−6) and palmitic (16:0) acids versus the above‐mentioned study in which oleic (18:1 n−9) (18.05%–19.71%), α‐linolenic (18:3 n−3) (13.81%–15.87%), and linoleic (18:2 n−6) (11.24%–21.55%) acids were the dominant fatty acids.

### 3.2 Fatty acid composition of coconut oil

As indicated above, there was a notable difference as concerns the fatty acid content and composition of the investigated microalgal powders. The amount of fat in *C. pyrenoidosa* powder was 3.4‐fold higher than that of *A. platensis* (Table 1) and comprised mostly of unsaturated fatty acids (65.36%) (Table 2). Nonetheless, the total fat content was quite low in both microalgae powders and as previous studies have already established that the availability of lipids in the meal is a critical factor that affects carotenoid bioaccessibility (Hornero‐Méndez & Mínguez‐Mosquera, 2007; Lemmens et al., 2014; O'Connell et al., 2008; Xavier et al., 2014), and an additional lipid source was employed. In this regard, organic cold‐pressed coconut oil was selected on account of its high content of saturated fatty acid and absence of carotenoids (Dauqan et al., 2011). Coconut oil is commonly added in smoothie recipes in combination with *Spirulina* or *Chlorella* powders for an extra source of energy. Some studies also suggest that the consumption of coconut oil has a positive impact on obese men and women (Assunção et al., 2009; Oliveira‐De‐Lira et al., 2018; Valente et al., 2018; Vogel et al., 2020), promoting weight loss of abdominal adiposity and less appetitive responses. Coconut oil is an unusual edible oil, comprised mainly of medium‐chain saturated fatty acids (C8–C12) and containing lauric acid (12:0) as dominant fatty acid (Pehowich et al., 2000). The fatty acid profile of the organic cold‐pressed coconut oil used in the current investigation (Table 3) was in accordance with literature data (Bhatnagar et al., 2009; Orsavova et al., 2015). In a recent study, pure coconut oil was obtained from coconut pulp and the fatty acid composition was examined (Pizzo et al., 2019). Similar to our results, lauric acid (12:0) was the major fatty acid (49.89 ± 1.08%), followed by myristic acid (14:0) (19.80 ± 0.73%) and comparable amounts of palmitic (16:0) (8.41 ± 0.74%) and caprylic (8:0) (7.19 ± 0.78%) acids. The most relevant characteristics of coconut oil in this particular study are the high percentages of saturated fatty acids (SFA, 96.15%) and medium‐chain fatty acids (MCFA, 68%).

**TABLE 3:** Fatty acid composition of coconut oil

Fatty acid	% of Total fatty acids^a^
Caproic acid (6:0)	0.55 ± 0.14
Caprylic acid (8:0)	10.51 ± 0.43
Capric acid (10:0)	6.14 ± 0.53
Lauric acid (12:0)	50.79 ± 1.55
Myristic acid (14:0)	18.69 ± 0.36
Palmitic acid (16:0)	6.87 ± 0.43
Stearic acid (18:0)	2.48 ± 0.25
Oleic acid (18:1 n−9)	3.41 ± 0.23
Linoleic acid (18:2 n−6)	0.44 ± 0.15
Arachidic acid (20:0)	0.11 ± 0.03
Σ SFA	96.15 ± 3.73
Σ MUFA	3.41 ± 0.23
Σ PUFA	0.44 ± 0.15
Σ MCFA	68 ± 2.65
Σ LCFA	32 ± 1.46

^a^ Mean ± *SD* (*n* = 3). MCFA represents medium‐chain fatty acids (C6–C12); LCFA represents long‐chain fatty acids (C14–C24); SFA represents saturated fatty acids; MUFA represents monounsaturated fatty acids; PUFA represents polyunsaturated fatty acids.

### 3.3 In vitro carotenoid bioaccessibility of *A. platensis* and *C. pyrenoidosa*

As mentioned previously, the bioaccessibility of carotenoids from the two microalgal supplements was assessed through an international standardized static in vitro digestion method (Minekus et al., 2014). Since other studies on carotenoid bioaccessibility from microalgae used different in vitro digestion protocols, a straight comparison of results is not feasible. To the best of our knowledge, there is only one available publication on carotenoid bioaccessibility from microalgae (*Nannochloropsis* sp.) that employed the same simulated digestion model (Bernaerts et al., 2020), with the omission of the oral phase.

#### 3.3.1 Bile source influence on carotenoid bioaccessibility

Various bile extract sources have been used in publications employing the standardized in vitro digestion model (Petry & Mercadante, 2019; Rodrigues et al., 2017; Wen et al., 2018). Here, we compared the use of porcine bile extract to that of bovine bile extract so as to achieve a final concentration of 10 mM in the final digestion mixture, as suggested by Minekus et al. (2014).

**FIGURE 3:**
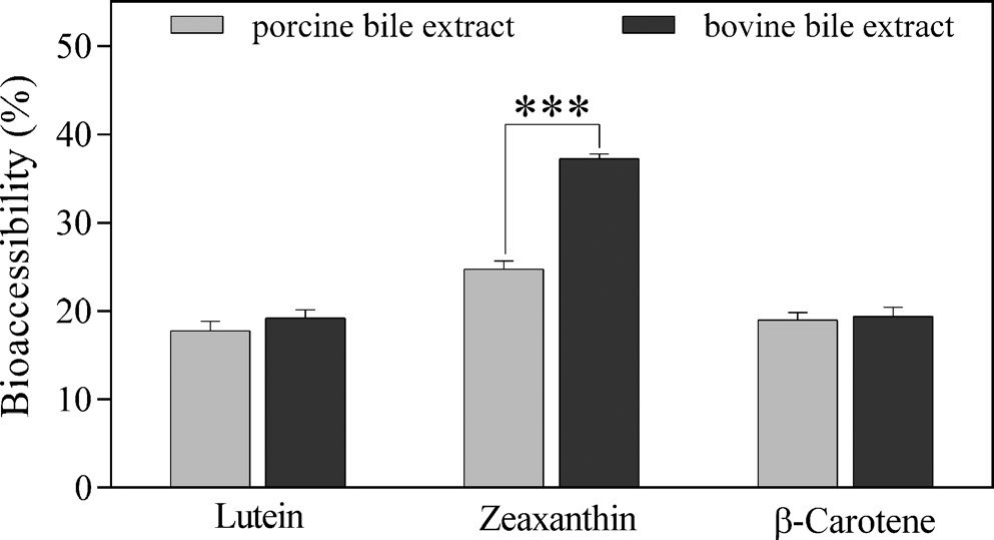
In vitro bioaccessibility (%) of lutein from *Chlorella pyrenoidosa* and of zeaxanthin and β‐carotene from *Arthrospira platensis* after the in vitro digestion using porcine bile extract as against bovine bile extract. Values are gives as mean ± *SD* (*** extremely significant *p* < .001)

No statistical significance was observed in the bioaccessibility of lutein from *C. pyrenoidosa* and in that of β‐carotene from *A. platensis* (from 17.77% to 19.19% and from 18.94% to 19.35%, respectively) after the replacement of the porcine extract with the bovine extract. More interestingly, a statistically significant enhancement was obtained as regards the bioaccessibility of zeaxanthin from *A. platensis* (from 24.68% to 37.24%) (Figure 3). In a similar manner, Wen et al. (2018) tested both sources of bile extract and higher zeaxanthin dipalmitate hydrolysis and bioaccessibility (from 15.9% to 29.5% and from 20.8% to 27.4%, respectively) were achieved when using the bovine bile extract. The authors highlighted an important aspect that may impact mixed micelles formation, namely the different granularity of the commercial products, bovine bile extract appearing as a fine powder, whereas porcine bile extract consisting of bigger and uneven particles. Also, Chitchumroonchokchai et al. (2004) observed a significant increase (from 27% to 49%) in carotenoid bioaccessibility from spinach puree after replacing the porcine bile extract with a mixture containing glycodeoxycholate, taurodeoxycholate, and taurocholate, three acids present in the human bile. Bovine bile extract appears to be a better candidate for mimicking the in vitro digestion conditions than porcine bile extract due to its similarity in bile acid composition to human duodenal contents as regards the main molecular species (Capolino et al., 2011).

In a previous study, the bioaccessibility of zeaxanthin from *Spirulina* was 4.93% and that of lutein from *Chlorella* 0.53% but the in vitro digestion conditions were not entirely provided and the investigated *Chlorella* strain was not mentioned throughout the study (O'Sullivan et al., 2011). In the current study, the higher bioaccessibility of zeaxanthin from *A. platensis* (37.24%) could be explained by its facilitated release from the easily digestible cell wall (composed of proteins and peptidoglycans) and by the presence of a higher saturated fatty acid content in lipid composition (Table 2). Yu et al. (2012) observed an increase of zeaxanthin concentration in human serum from 0.06 to 0.15 μmol/L after ingesting a single dose of *Spirulina*, emphasizing not only the high bioaccessibility but also the high bioavailability of zeaxanthin from this source.

The bioaccessibility of zeaxanthin from *A. platensis* was greater than that of β‐carotene (37.24% as against 19.35%). This higher capacity of polar xanthophylls such as lutein and zeaxanthin to accumulate in the micellar fraction was also observed in other studies (Chitchumroonchokchai et al., 2004; Kaulmann et al., 2016; O'Connell et al., 2008; Tudor et al., 2020) and could be explained by their higher solubility compared to apolar carotenes such as β‐carotene. Being less hydrophobic, the location of xanthophylls in stomach lipid globules appears to be closer to the surface monolayer (along with proteins, phospholipids, and partially ionized fatty acids) as opposed to that of carotenes which reside inside the triacylglycerol‐rich core (Canene‐Adams & Erdman, 2009). Thus, due to their position, xanthophylls are more readily incorporated into mixed micelles in the duodenum than the highly hydrophobic carotenes.

In the case of *C. pyrenoidosa*, because of its thicker cell wall compared to other *Chlorella* strains (Duan et al., 2017), the disruption of the cellulose‐rich walls during the drying process was specifically mentioned on the label. This is an important aspect as wall breakdown has been proven to contribute significantly to the enhancement of lutein bioaccessibility from *Chlorella vulgaris* (from 26% to 57% and 73% after microfluidization at two different pressures) (Cha et al., 2011). Nevertheless, more details regarding the processing method were not mentioned on the label; therefore, it is not possible to assess the impact of cellular breakdown on lutein bioaccessibility. A comparable value for lutein bioaccessibility (18%) was obtained by Gille et al. (2015) following the in vitro digestion of *Chlorella vulgaris* after sonication for 15 min.

#### 3.3.2 Lipophilic phase influence on carotenoid bioaccessibility

**FIGURE 4:**
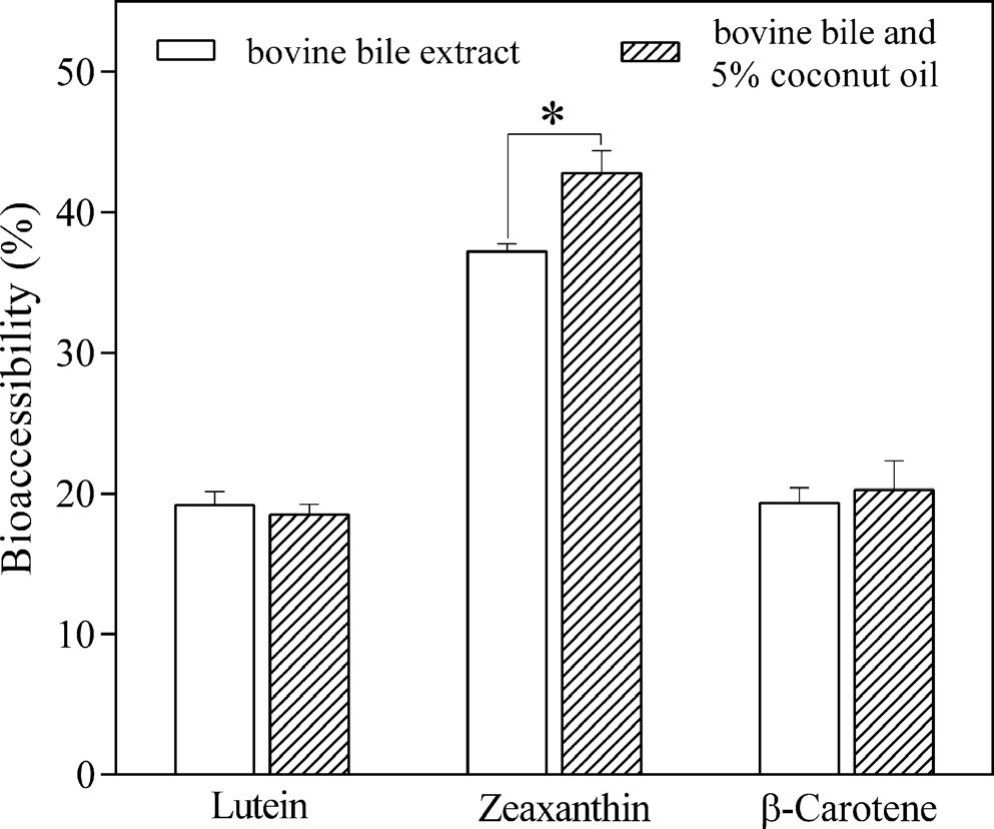
In vitro bioaccessibility (%) of lutein from *Chlorella pyrenoidosa* and of zeaxanthin and β‐carotene from *Arthrospira platensis* after the in vitro digestion using bovine bile extract and with bovine bile extract along with 5% coconut oil. Values are gives as mean ± *SD* (* significant *p* < .05)

Upon addition of 5% coconut oil in the in vitro digestion of the two microalgal powders, a similar pattern to that obtained for bile source evaluation was observed, with a statistically significant increase in the case of zeaxanthin bioaccessibility from *A. platensis* (from 37.24% to 42.82%) (*p* < .05). Concerning the other major carotenoids, the increase in β‐carotene bioaccessibility (from 19.35% to 20.29%) from *A. platensis* was not statistically significant, as well as the slight decrease in lutein bioaccessibility from *C. pyrenoidosa* (from 19.19% to 18.48%) (Figure 4).

Against our expectations, the addition of a different source of lipids had no significant effect on lutein bioaccessibility, which may suggest that the amount of lipids already existing in the *C. pyrenoidosa* powder was sufficient for the release of lutein, or that other parameters had a much more predominant role in influencing lutein bioaccessibility than lipids. Previous studies have emphasized the negative impact of an increased amount of dietary fiber (Aschoff et al., 2015; Yonekura & Nagao, 2009) and of a higher degree of unsaturation in lipid composition (Gleize et al., 2013; Yuan et al., 2018) on xanthophyll bioaccessibility. It appears that the unknown level of cellular breakdown during the drying process of the microalgal biomass, along with the high amount of dietary fiber (twofold higher than that of *A. platensis*) and of unsaturated fatty acid content (65.36% as against 38.92% for *A. platensis*), had a considerable influence in the overall bioaccessibility of lutein. In contrast, the absence of indigestible cellulose in *A. platensis* cell wall, together with a lower amount of both dietary fiber and unsaturated fatty acids, yielded a better zeaxanthin bioaccessibility from *A. platensis*, which seemed to be further enhanced by supplementation with coconut oil. The commercial supplement of *A. platensis* in powder form is added as a minor ingredient in meals, whereas that in tablet form is usually ingested between meals along with water. Based on this study, it could be presumed that the ingestion of *A. platensis* supplement in powder form would enable even a greater zeaxanthin bioaccessibility than in tablet form, considering that meals (such as smoothies, salads, yogurt, sauces, or snacks), in contrast to tablets, contain at least a trace amount of lipids that could promote zeaxanthin bioaccessibility.

Other authors have included coconut oil in their studies in order to evaluate its effect on carotenoid bioaccessibility. Yuan et al. (2018) reported an increase in lutein bioaccessibility from 20.9% to 46.5% after mixing spinach puree with an excipient emulsion containing coconut oil and in another study the bioaccessibility of zeaxanthin from goji berries increased from 6.7% to 13.3% after the addition of 1% coconut oil (Hempel et al., 2017). The beneficial impact of medium‐chain saturated fatty acids on xanthophyll bioaccessibility could be explained by the formation of smaller mixed micelles in the small intestine, which consequently implies a greater surface area for the more polar carotenoids to be incorporated (Yuan et al., 2018). By contrast, bigger mixed micelles formed after the digestion of long‐chain polyunsaturated fatty acids provide a larger hydrophobic core and are therefore more favorable for the incorporation of nonpolar carotenoids such as β‐carotene (Zhang et al., 2015). This could also explain our insignificant increase in β‐carotene bioaccessibility after the addition of coconut oil.

**FIGURE 5:**
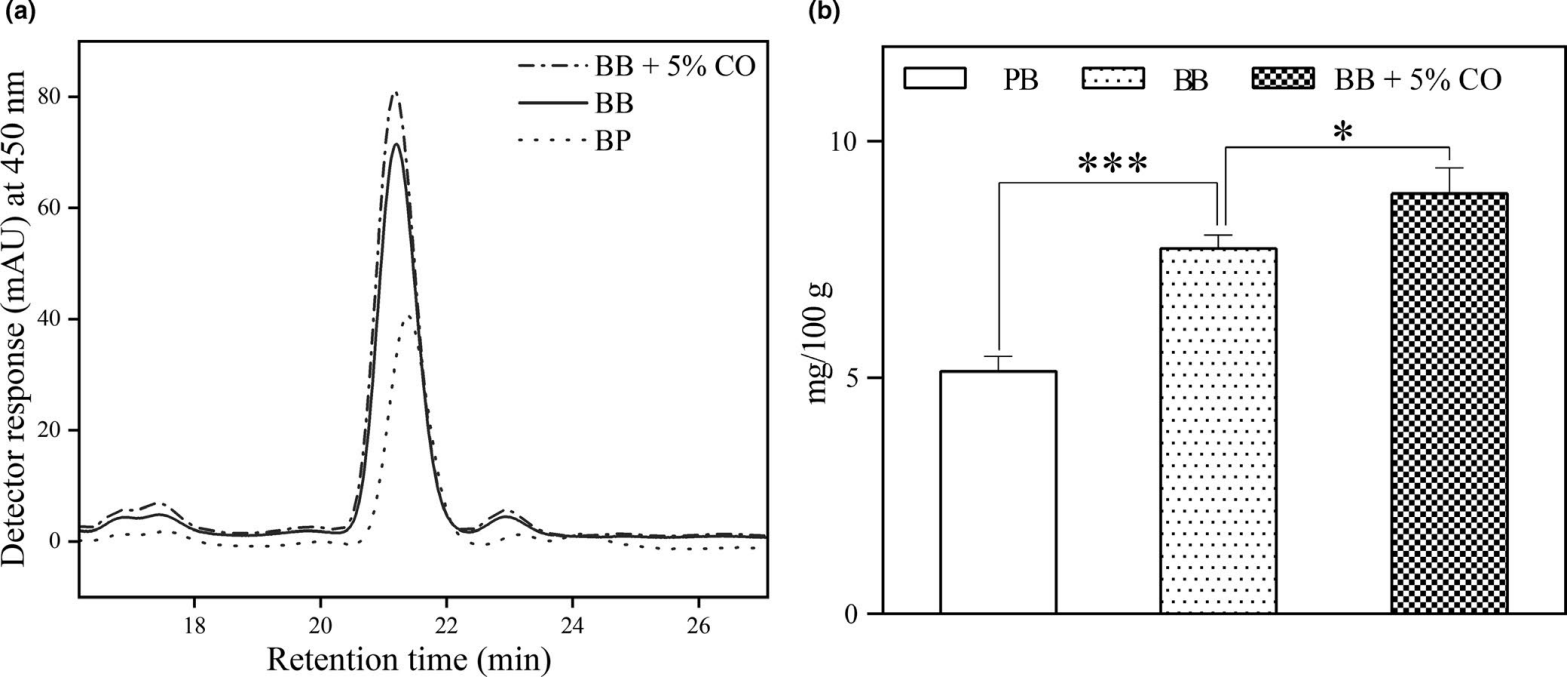
Zeaxanthin signal (a) and micellar concentration (mg/100 g) (b) after the in vitro digestion of *Arthrospira platensis* using porcine bile extract (PB), bovine bile extract (BB), and bovine bile extract along with 5% coconut oil (BB + 5% CO). Values are gives as mean ± *SD* (* significant *p* < .05, *** extremely significant *p* < .001)

One of the most important findings of this study was the high increase in zeaxanthin response after the in vitro digestion of *A. platensis* in different conditions (Figure 5a), and for this reason, special emphasis will be placed on this matter. On account of their accumulation in the retina, lutein and zeaxanthin are collectively known as the “macular pigments” (Krinsky et al., 2003), and among these two oxygenated carotenoids lutein is more abundant in nature and has been the focus of a larger number of studies compared to its structural isomer. Not only is lutein occurrence in natural food sources higher, but also its content and bioaccessibility (Tudor & Pintea, 2020). Consequently, research on plentiful and bioaccessible food sources of zeaxanthin other than the few already confirmed (Hempel et al., 2017; Tudor et al., 2020) is of great scientific interest. Apart from being an easily digested food matrix, one of the most important advantages of *A. platensis* as a source of zeaxanthin is represented by the deposition form of this xanthophyll. Zeaxanthin appears to be mostly present in its free form in microalgae (Cha et al., 2012; Granado‐Lorencio et al., 2009), more readily available for micellization and intestinal absorption than from zeaxanthin‐rich plant sources, in which case additional enzymatic cleavage of zeaxanthin mono‐ and diesters to free zeaxanthin during digestion is required.

In this study, zeaxanthin concentration in the micellar phase was gradually increased after the simulated digestion with the use of porcine bile extract, bovine bile extract, and after the addition of a dietary lipid rich in medium‐chain saturated fatty acids (5.1 mg/100 g, 7.7 mg/100 g and 8.9 mg/100 g, respectively) (Figure 5b). These values represent the actual amount of zeaxanthin transferred from the food matrix (i.e., dry microalgal biomass) into micelles that become available for absorption by the enterocytes. These data further translate into percentage of bioaccessibility and when compared to the values obtained by other research groups employing the same simulated digestion protocol, the current results seem above the average. Zeaxanthin bioaccessibility from *A. platensis* after the digestion with bovine bile extract and coconut oil (42.82%) was considerably higher than from several plant‐based sources such as astringent persimmon (2.5%) (Cano et al., 2019), goji berries (13.3%) (Hempel et al., 2017), *Pouteria lucuma* fruit (1.6%–5.8%) (Gómez‐Maqueo et al., 2020), and from some maize‐based food formulations (boiled kernels, porridge and tortilla; 2.4%, 7.8%, and 18.4%, respectively) (Zhang et al., 2020) but lower than that obtained from hard‐boiled egg yolk (90%) (Rodrigues et al., 2017). As regards, the other publication on carotenoid bioaccessibility from microalgae (*Nannochloropsis* sp.) involving the same simulated digestion protocol (Bernaerts et al., 2020), zeaxanthin bioaccessibility, was higher than from the untreated and high‐pressure homogenized suspension of *Nannochloropsis* sp. (9% and 19%, respectively) but similar to that obtained from an oil‐in‐water emulsion prepared with the extracted *Nannochloropsis* sp. oil (54%).

## 4 CONCLUSIONS

Carotenoid consumption has numerous benefits on human health. Several microalgae species can produce and accumulate carotenoids, especially β‐carotene and xanthophylls. Considering the increase in population awareness of disease prevention through diet and the constant demand for natural rather than synthetic, microalgae will undoubtedly become an important food source of carotenoids and other valuable bioactive compounds in the future and investigation not only into their beneficial impact upon human health, but also into their gastrointestinal fate constitutes a valuable field of research.

In summary, the use of the bovine bile extract instead of porcine bile extract in the in vitro digestion of the two microalgal supplements improved carotenoid bioaccessibility to a different extent and the addition of 5% coconut oil led to a significant increase in the bioaccessibility of zeaxanthin from *A. platensis* (from 37.2% to 42.8%). Even though many carotenoid‐rich food sources are known, the fraction of these lipophilic compounds that becomes accessible for absorption is fairly low due to their restricted release from the food matrix. Based on our findings, microalgal powders could serve not only as a potential alternative for animal proteins, but also as a more‐bioaccessible source of high‐added value natural compounds such as carotenoids. The unpretentious cultivation conditions along with the faster growing rate render microalgae a suitable food source for carotenoid production. Furthermore, in comparison with higher plants, large‐scale cultivation of microalgae is not restricted to a certain period of the year and does not require arable land or burdensome operations.

## CONFLICT OF INTEREST

The authors declare that they do not have any conflict of interest.

## Data Availability

The data that support the findings of this study are available from the corresponding author upon reasonable request.
